# Developing patient-centered outcomes research infrastructure in a rural community through patient and stakeholder engagement and education during the COVID-19 pandemic

**DOI:** 10.1017/cts.2022.486

**Published:** 2022-10-28

**Authors:** Carly Lovelett, Michelle Medeiros, Daniel Jaremczuk, Jennie Flanagan, Jennifer Shaver, Elaine LaLone, Eyal Kedar

**Affiliations:** 1 Department of Clinical and Rural Health Research, St Lawrence Health, Potsdam, NY 13676, USA; 2 The PATIENTS Program, University of Maryland School of Pharmacy, Baltimore, MD, USA; 3 Community Partner; 4 Patient Partner; 5 Division of Rheumatology, St Lawrence Health, Potsdam, NY 13676, USA

**Keywords:** Patient-centered outcomes research, rural health, virtual education, clinical research infrastructure, patient engagement, community engagement

## Abstract

In addition to facing numerous healthcare disparities, rural America is chronically underrepresented in clinical research. This gap was made more evident during the COVID-19 pandemic. St Lawrence Health, located in rural Upstate New York, established its Clinical and Rural Health Research Department in 2015 to help close this gap. The research department then launched the DISRUPTS (Developing InfraStructure for Research to Utilize Patient-centered Techniques at St Lawrence Health System) program to build the infrastructure to conduct Patient-Centered Outcomes Research (PCOR). Together with a diverse committee, the team used proven methods and frameworks to develop a model for engagement, content creation, and education delivery that was successfully used to create educational programs on PCOR and COVID-19. The resulting DISRUPTS webinars had a combined total of over 450 live attendees and over 1,110 views on recordings. Furthermore, nearly one-third of those who participated in the COVID-19 vaccines webinar indicated they were more likely to receive a COVID-19 vaccine after taking part. DISRUPTS can serve as an important model for other rural communities that aim to increase access to and engagement in PCOR, and which hope to improve outreach and education efforts in their communities.

## Introduction

Although 15% of the US population lives in rural regions, rural Americans continue to face a multitude of disparities compared to urban Americans [1]. In addition to disparities in social determinants of health (economic instability, limited access to healthy food and transportation, etc.) [2], there is also a well-documented shortage of physicians[3] and, though less often discussed, a near absence of clinical research infrastructure and expertise in rural America [4]. These disparities were further exposed at the onset of the COVID-19 pandemic, during which many treatment options were only available in the context of clinical trials [5]. Rural patients are on average older, poorer, less healthy, less health literate, and more likely to die from COVID-19 than their urban counterparts [2,6]. Therefore, rural communities in the USA are in critical need of education on infection prevention and mitigation strategies, as well as additional initiatives to connect these populations to clinical research opportunities.

Rural St Lawrence County (SLC) is New York (NY) State’s largest county by area and one of its poorest. With a population of just over 100,000 people, SLC has a median per capita income of $26,676^7^. The Clinical and Rural Health Research Department (“research department”) of St Lawrence Health (SLH) [8] was established in 2015 to provide patients in SLC with local access to clinical trials for the first time and to help address rural health disparities.

In 2019, the research department launched the DISRUPTS (Developing InfraStructure for Research to Utilize Patient-centered Techniques at St Lawrence Health System) program. Funded through a Eugene Washington Patient-Centered Outcomes Research Institute® (PCORI®) [9] Engagement Award, DISRUPTS aimed to incorporate the voices of patients and other stakeholders in addressing two fundamental and nearly ubiquitous barriers to conducting Patient-Centered Outcomes Research (PCOR) in rural America: a lack of research infrastructure and a lack of research knowledge/awareness. In response to the global COVID-19 pandemic, the goals of DISRUPTS later expanded to include providing education on COVID-19.

To collaboratively guide the project forward, the research team established a diverse Stakeholder Advisory Committee (SAC). As defined by the SAC, PCOR is when researchers work with patients, caregivers, and other stakeholders to decide what health-related questions are important, and the best ways to research these questions and share the results. Patient centeredness is best achieved by engaging patients and key stakeholders in every step of the research process, and ensuring meaningful engagement by incorporating components like building reciprocal relationships, colearning, reassessment, and feedback [10]. These engagement strategies foster patient and stakeholder ownership in the research process; this ownership can lead to not only more applicable and trusted research results, but also more successful research conduct and change implementation [11].

Through DISRUPTS, the research team and the SAC adapted the 10-step framework for continuous engagement [12] and applied it to stakeholder engagement, content creation, education delivery, and program evaluation. This same model was then successfully reapplied to the COVID-19 educational program. This DISRUPTS model can serve as a template for other rural regions to engage stakeholders, provide community education, and build patient-centered research infrastructure.

## Materials and Methods

As an educational project, DISRUPTS was determined to be exempt from Institutional Review Board (IRB) oversight by the St Lawrence Health IRB. Survey and feedback data were collected using the following platforms: Qualtrics, Zoom, and Google Forms. Quantitative analysis was completed using Microsoft Excel.

### Formation of Stakeholder Advisory Committee

Prior research has demonstrated that well-intended efforts by healthcare organizations to be patient-centered often rely on misguided assumptions of what patients want, which in turn leads to patient disengagement and unsustainable change [13]. To combat this disengagement, DISRUPTS was structured around proven engagement principals and training evaluation frameworks, including the 10-step framework for continuous engagement [12], factors of successful rural engagement [14], and the Kirkpatrick model of evaluation [15].

The first step to ensure continuous engagement was to create the SAC. The SAC was a nine-member group that consisted of local patients, past research participants, clinicians, nurses, and community members with diverse backgrounds and experiences. The membership selection process involved the DIRSUPTS writing team (i.e., research department, medical lead, and patient/community advocates) identifying and narrowing down a large list of interested individuals, and then voting on the committee composition that best represented broad community domains (e.g., included a local farmer and a professor from a local university).

Monthly meetings, facilitated by the SLH Director of Research, were attended by the SAC, the research department, and an SLH executive for institutional support. Additionally, representatives from the PATIENTS Program at the University of Maryland, Baltimore School of Pharmacy [16] attended meetings to provide subject–matter expertise in stakeholder engagement and PCOR.

During the DISRUPTS kick-off meeting, as well as throughout the duration of the project, a deliberate focus was placed on incorporating the six factors of successful rural engagement: building relationships, defining expectations, establishing communication guidelines, developing shared understanding, facilitating dialog, and valuing contributions [14]. These factors where interweaved into a codeveloped group charter document, which outlined the mission, roles, and responsibilities, objectives, etc. associated with the DISRUPTS project. In addition to these factors, a key aspect of our engagement strategy was to foster ownership; the members of the SAC were motivated to meet or exceed defined expectations, because they felt ownership in the process and the results.

The 10-step framework for continuous engagement outlines 10 steps for patient/stakeholder engagement throughout a project’s lifecycle, categorized under three main phases: planning, doing, and delivering [12]. These phases of the DIRSUPTS project are outlined below.

### Planning

#### Surveying the Population

To identify our rural community’s specific needs and interests, the SAC codeveloped and distributed a survey designed to determine baseline understanding and interest levels in learning about PCOR, the preferred methods for education delivery, and topics of interest. A concerted effort was made to gather input from harder-to-reach populations, leading to a multifaceted survey distribution, which included emailing specific individuals and groups, distributing paper copies at health fairs and other in-person events/community locations, social media outreach, and internal SLH email distribution. By directly surveying the community on what they wanted to gain from a PCOR educational program, we were spreading awareness of the program while also giving the community ownership over the final product. The resulting data were reviewed and used to develop a PCOR educational series that would address the needs of the community and would be suitable for individuals with all backgrounds. In light of the COVID-19 pandemic, this series was ultimately delivered virtually rather than in-person.

### Doing

#### Content Development and Delivery

A three-part webinar series, titled “Learning Together, Leading Together: Shaping Patient-Centered Research in the North County,” was cocreated to address the topics of interest from the community survey. The group broke into subcommittees for each topic and then took an iterative approach to content creation until the SAC approved the presentations.

Having to adapt to an online environment, special care was taken to ensure appropriate, effective and engaging educational design, and delivery [17]. The SAC worked together to create content that was accessible to nonscientific audiences, but still interesting and engaging for those with scientific/medical expertise. Webinars included aspects of direct lecture, original videos, sourced videos, personal stories, and discussion/question and answer segments. Webinars were scheduled monthly via Zoom videoconferencing and were codelivered by at least one research department member and a community/patient partner. Invitations to attend live sessions were widely disseminated to the community and SLH via word of mouth, social media, radio, email, and press releases.

Key topics covered in each part of the webinar series are below:

- Part 1: Introduction to and examples of patient-centered outcomes research
- Part 2: Research methods and engagement principles
- Part 3: Key considerations for engaged research and the importance of PCOR and community involvement

#### Attendee Engagement and Feedback

Polling and knowledge checks were integrated into all PCOR webinars to increase attendee engagement and assess knowledge acquisition. A standardized list of feedback questions was presented at the end of sessions, asking attendees to rate them on a four-point Likert scale (Response options: Extremely, Very, Moderately, and Slightly) according to four questions (How informative was this session?; How likely are you to attend similar events in the future?; How likely are you to talk about what you learned today with others?; and How enjoyable was this session?). Additionally, a link to a survey with open-ended questions was sent to attendees for qualitative feedback. The surveys were approved by the SAC and all had a Flesch-Kincaid Grade Level of less than eighth grade [18]. After each webinar, the SAC reviewed all feedback in order to incorporate it into and improve upon subsequent webinars.

### COVID-19 Education: Planning and Doing Phases

In response to the global COVID-19 pandemic and the concomitant national and local increases in depression, anxiety, and substance abuse [19], the team also developed a three-part live webinar series and a five-part prerecorded series addressing important topics around COVID-19. The DISRUPTS COVID-19 education program replicated the successful model of community engagement, content development, and delivery used for the PCOR education series as outlined below:

#### Planning:

1. Formed a COVID-19-specific stakeholder group (“COVID-19 Committee”), which included COVID-19 survivors, clinicians who were actively treating COVID-19, and community members to provide perspectives and expertise alongside the SAC
2. Surveyed the community to understand interests, needs, and questions

#### Doing:

1. Codeveloped educational content based on community survey responses
2. Launched a multifaceted outreach campaign
3. Delivered the educational webinars via Zoom. Webinars were presented by local experts (e.g. healthcare providers, social workers, director of public health).
4. Solicited feedback from attendees to improve upon future webinars

The complete list of webinars is provided below:

- COVID-19: fact versus fiction
- COVID-19 in rural America
- COVID-19 vax versus fiction: answering questions and presenting the facts about COVID-19 vaccines
- COVID-19 and mental health/substance use series (prerecorded):
  - The impact of COVID-19 on substance use
  - Navigating COVID-19 and the impact on children and families
  - COVID-19 and school
  - COVID-19 and mental health
  - St. Lawrence County COVID-19 response: a year in review

### Delivering: Dissemination

Recordings of the PCOR and COVID-19 webinars were finalized and uploaded to the research department webpage for further viewing and sharing [8]. In addition to press releases and emails blasts, members of the SAC and COVID-19 Committee also distributed the webinars amongst their social and professional networks. For example, one member of the SAC who is the superintendent of a local school district distributed these resources via his weekly newsletter to faculty and families. Lastly, a codeveloped infographic with program results was mailed to each member of the SAC and emailed to program attendees and SLH employees.

## Results

### Community Survey Results

190 individuals responded to the preliminary PCOR community survey, including representation from patients, caregivers, healthcare providers, SLH employees, and community members. Of the responses, 64% had never heard of PCOR previously, and 71% indicated that they would or might be interested in participating in a training program on the topic. Respondents indicated a preference towards in-person learning rather than virtual, and multiple shorter sessions instead of one long one. The top three topics of interest were 1) A basic definition and history of patient-centered outcomes research, 2) Examples of patient-centered outcomes research projects, and 3) How patient-centered outcomes research findings are applied in healthcare settings.

There were 69 respondents to the COVID-19 community survey, including representation from patients, caregivers, healthcare providers, and students. Although 77% of respondents rated themselves as extremely comfortable using measures to prevent the spread of COVID-19, only 23% rated themselves as extremely comfortable assessing the risk of participating in social activities during the pandemic. Respondents submitted 25 written questions/topic requests for the educational sessions. These ranged from “Do you have to wipe off groceries and packages before entering your home?” to “What are the long term risks of the vaccine and the long term impacts of having COVID?” to “Dealing with mental fatigue.”

### Intervention Results

**Fig. 1.**
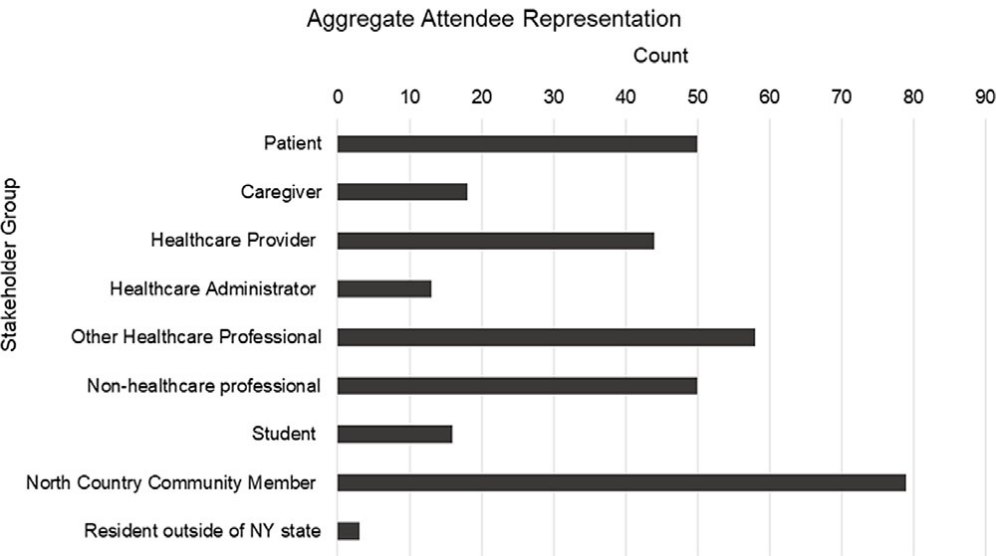
Aggregate attendee representation. Attendees’ self-identification of stakeholder group(s) they belong to. Responses are aggregated across all patient-centered outcomes research (PCOR) and COVID-19 live webinars (*N* = 331).

As seen in Fig. 1, the PCOR and COVID-19 education sessions were attended by individuals from many backgrounds. The three-part PCOR series had 118 live attendees and has generated 223 views on the recordings (as of October 4, 2022) and the COVID-19 series had 340 attendees at the live events with 890 views of the recordings.

Effort was made to evaluate the two educational programs at each level of the Kirkpatrick Model of Training Evaluation (i.e. Reaction, Learning, Behavior and Results) [15], summarized below.

#### Reaction: How did participants respond to the training?

**Fig. 2.**
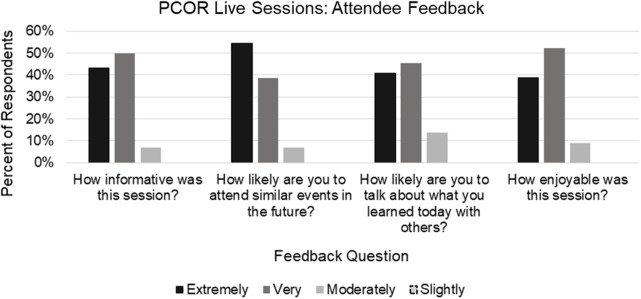
Patient-centered outcomes research (PCOR) live sessions: attendee feedback. Aggregate attendee feedback responses across the three PCOR webinars (*N* = 44).

Of the 44 attendees who responded to the PCOR feedback questionnaire (37% response rate), 91% indicated that the sessions were either “very” or “extremely” enjoyable (Fig. 2). There were also high percentages of “very” and “extremely” responses for the likelihood of attending similar events in the future (93%), likelihood of talking about what they learned with others (86%), and on the session being informative (93%) (Fig. 2).

**Fig. 3.**
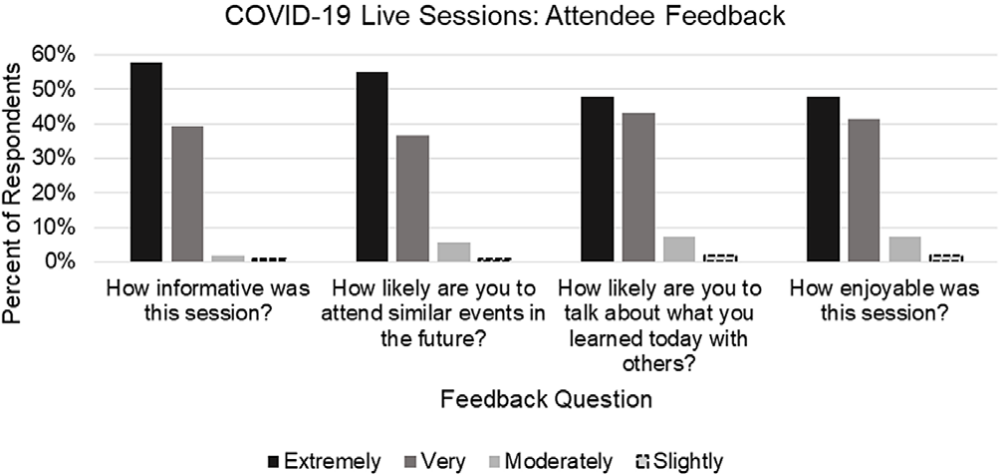
COVID-19 live sessions: attendee feedback. Aggregate attendee feedback responses across the three live COVID-19 webinars (*N* = 109).

For the COVID-19 program, 97% of the 109 respondents to the feedback questionnaires (32% response rate) indicated that the webinars were either “very” or “extremely” informative (Fig. 3). The full breakdown of feedback responses is in Fig. 3, with 89% or higher indicating “very” or “extremely” for each question.

Another key part of the “reaction” level evaluation was qualitative feedback. Below is a representative selection of responses to the open-ended feedback questions across all the PCOR and COVID live webinars:

**Question:** What was your favorite/the most valuable part of the seminar?

Answers:

- PCOR Webinars:
  - “I enjoyed hearing the personal stories about how research can be made more patient centered”
  - “How the North Country can get involved; not just large cities.”
  - “I got the most from hearing people tell of the advantages of agreeing to participate in a research study.”
- COVID-19 Webinars:
  - “The entire presentation was engaging and informative. I plan on sharing the video with my friends and family when it is available.”
  - “Free to the public”
  - “I liked that this was down to earth- I didn't need medical degree to understand”

**Question:** What suggestions do you have for improvement?

Answers:

- PCOR Webinars
  - “Condense the intro.”
  - “The brief video had an excellent message, but there was a lot of background noise. If a better option were available, I would elevate the experience with a clearer message.”
  - “Right now, not many! Of course it would be great to have this type of seminar in person, but via zoom makes more sense at this time.”
- COVID-19 Webinars
  - “I was unable to see slides etc. But I have a tablet and am new to Zoom. I was glad the presentation wasn't dependent on those visuals.”
  - “please consider awarding contact hours or certificate of attendance”
  - “It was just right. Thank you for providing the webinar. It was very informative.”

#### Learning: How well did participants acquire the intended knowledge/skills?

**Fig. 4.**
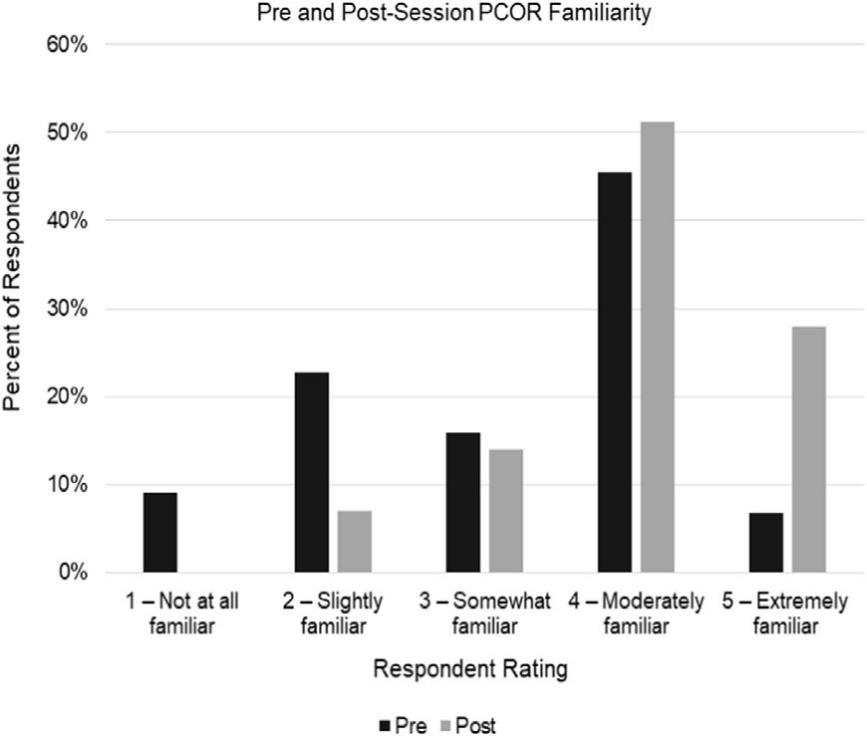
Pre and post-session patient-centered outcomes research (PCOR) familiarity. Self-reported PCOR familiarity based on attendees’ responses at the beginning and end of each webinar. Responses were aggregated across the three PCOR sessions (NPre = 44; NPost = 43).

Based on in-session polling, attendees demonstrated a higher self-reported familiarity with PCOR after attending the sessions when compared to baseline (Fig. 4). Specifically, 79% of respondents indicated that they were “moderately” or “extremely” familiar with PCOR after attending the session compared to 52% at baseline, and only 7% indicated they were “not at all familiar” or “slightly familiar” after attending compared to the baseline of 32%. This shift in familiarity with PCOR is illustrated in Fig. 4 and is supported by a 93% correct response rate on content-testing questions at the conclusion of the sessions compared to 85% correct response rate at baseline.

Due to the rapidly evolving COVID-19 landscape and the use of local experts to deliver COVID-19 sessions, there were no content-testing questions included in the COVID-19 webinars. However, general feedback from the open-ended questions offered anecdotal evidence that attendees left with a greater understanding of the topics. For example, one attendee said that their favorite/most valuable part of the seminar was “It was just good information that I did not all know before,” and another stated that they valued learning about “how the vaccine works; why current vaccines are safer than 20 yrs ago; why some people have allergic reactions; FDA approval process.”

#### Behavior: How have participants applied their acquired knowledge/skills?

Ninety percent of respondents across all webinars indicated that they were “extremely” or “very” likely to talk about what they learned with others. If we extrapolate this percentage to all live attendees and viewers of recorded content (*N* = 1571), we could estimate that we started over 1,400 new conversations about this content across the region and likely beyond.

Perhaps the most notable behavior-level indictor on this project was that 28% of survey respondents indicated they were more likely to get a COVID-19 vaccine after attending the live session on COVID-19 vaccines. As lower vaccination rates in many rural areas [20] are arguably the most important driver of the ongoing rural–urban COVID-19 mortality gap [21,22], this program demonstrates the potential of patient-centered education efforts to increase the trust of rural patients in the larger American healthcare system.

#### Results: What long-term outcomes resulted from the training?

DISRUPTS was the first known educational series on PCOR offered in SLC to date, triggering the beginning of structured community and patient-centered research in our rural Northern NY region. To ensure sustainability, the research team formed a patient-centered research focus group as one of the last actions of the DISRUPTS program. This group is made up of patient, caregiver, and community member volunteers who attended the DISRUPTS webinars. Members apply the knowledge gained through the educational sessions and work together to inform on potential research questions, research design, patient-facing documents, and dissemination activities. This focus group has been given ownership in the strategic direction of the clinical research department and has since led to the initiation/improvement of several ongoing rural-health focused PCOR projects and departmental processes.

Lastly, through the outreach and engagement work on the DIRSUPTS project, the research team and SLH at large established numerous lasting connections with community members, patients, and COVID-19 and health experts. This includes later hiring a SAC member as a clinical research nurse coordinator, who began her research career already trained on patient-centered research.

## Discussion

During the DISRUPTS program, a diverse group worked together to codevelop a unique program that successfully educated key stakeholders on conducting PCOR and how it can improve the health of our rural region, as well as on topics about COVID-19. Through this effort, DISRUPTS also spread awareness, understanding, and interest in research in general.

While efforts were made to offer the DISRUPTS webinars to all interested individuals in our region, we acknowledge that offering a solely virtual education opportunity will have excluded people in our area who have either no or limited access to the internet. This selection bias may have influenced the results in a way that made them not completely representative of the local population. As public safety measures relax, future efforts should include in-person delivery of the educational materials that were developed through DISRUPTS. Once completed, a combined and subgroup analysis of the virtual and in-person programs should be conducted to inform on future initiatives. Future iterations should also expand upon measurements to better assess the impact of the program (e.g. knowledge retention questions during the COVID-19 webinars, 3- and 6-month follow-up to quantitatively measure behavior change).

We require practical research to address the needs of rural America, and to achieve this the voices of patients must be considered and valued. While the DISRUPTS project has and will continue to make a meaningful impact on the rural Northern NY region, more work is needed to ensure patients and other stakeholders are engaged in and educated on the research process throughout rural America, and are given ownership in the direction that research takes. The resulting benefits of actively and meaningfully engaging patients and other stakeholders in the research process include improved relevance of study results to patients, improved research prioritization, better research recruitment and retention rates, and more effective dissemination of results [11,23,24].

Our model for content creation and delivery can serve as a template for future efforts to educate communities and develop clinical research infrastructure and trust in rural areas. Through efforts such as these, a larger and essential goal of developing networks of rural PCOR sites that generate and share data can be realized. The result of such networks will be a much clearer picture of the still largely undefined, under-resourced, and poorly understood world of healthcare in rural America. The path forward begins with projects like DISRUPTS and other completed and ongoing rural PCOR initiatives [14,25], which engage patients and community members in research, link those individuals with one another and with local and national health leaders, and give all members of rural communities a voice in both the research and healthcare strategies that affect their lives.
